# Comparative analysis of two cryopreservation systems of ovarian tissues in female Wistar rats

**DOI:** 10.5935/1518-0557.20140085

**Published:** 2014

**Authors:** Isabel C. L. O. Durli, Ana Helena R. Paz, Paula B. Terraciano, Eduardo P. Passos, Elizabeth O. Cirne-Lima

**Affiliations:** 1 Graduate Program in Veterinary Sciences - School of Veterinary - Universidade Federal do Rio Grande do Sul (UFRGS) - Porto Alegre, RS, Brazil; 2 Laboratory of Embryology and Cellular Differentiation - Experimental Research Center - Hospital de Clínicas de Porto Alegre - Porto Alegre, RS, Brazil; 3 Graduate Program in Medical Sciences - Faculdade de Medicina, UFRGS - Porto Alegre, RS, Brazil

**Keywords:** cryopreservation, dimethyl sulfoxide, ethylene glycol, ovarian follicle

## Abstract

**Objective:**

The aim of this study was to determine the most efficient protocol for cryopreservation of ovarian tissue using the automatic Freeze Control® system and to test two different cooling curves combined with two different cryoprotectants: dimethyl sulfoxide (DMSO) and ethylene glycol (EG).

**Methods:**

In this study, 20 female Wistar rats underwent bilateral oophorectomy. The ovaries were divided into two groups: one cryopreserved in 1.5M DMSO and the other in 1.5M EG. Two cooling curves, slow (1h 50min) and rapid (35min) were analyzed. Tissue samples were frozen, thawed, fixed, and stained with hematoxylin and eosin to analyze oocyte integrity. Follicular analysis was performed under optical microscopy (400x magnification) and preantral follicles were classified as primordial or primary according to developmental stage. ANOVA was performed, and Tukey’s test was used for comparison between means, with *P*<0.05 defined as significant.

**Results:**

In cryopreserved tissue, the follicles with preserved integrity in each ovary were 79% primordial and 29% primary. In non-frozen (control) tissue, all follicular types were observed (primordial, primary, secondary, preantral, and antral). Reversible changes included cytoplasmic vacuolization and irregular cell outline. Irreversible changes included pyknosis. EG was more efficient than DMSO, preserving a greater number of viable primordial and primary follicles. Comparison of both cooling curves revealed no statistically significant differences between them.

**Conclusion:**

The EG is more effective as a cryoprotectant than DMSO for obtaining higher viable numbers of primordial and primary follicles from rat ovarian tissue. Further studies are needed to demonstrate ovarian functionality, such as detection of hormone levels.

## INTRODUCTION

The survival rates of patients undergoing cancer treatment have been rising in recent years (Schuffner *et al*., 2004). However, surgical or clinical treatment modalities, including chemotherapy and radiation therapy, can impair fertility. Childhood cancer accounts for 1% of malignancies. Because of advances in major treatment, nearly 80% of children and adolescents who receive a diagnosis of cancer can now expect long-term survival (Binart & Sauvat, 2011, Donnez & Dolmans, 2013). In an attempt to minimize the negative side-effects of cancer treatment, cryopreservation of ovarian tissue has arisen as a promising technique for fertility preservation in young women (Courbiere *et al*., 2005, Ernst *et al*., 2010). Cancer treatments affect germ cells, which are highly susceptible to toxicity, to different extents; their main fertility-related complication is premature ovarian failure (Almodin *et al*., 2004, Aubard *et al*., 1996, Martinez-Madrid *et al*., 2004). Currently available methods of oocyte preservation still leave much to be desired; use of frozen oocytes is associated with very low pregnancy rates (Mazur *et al*., 1972, Porcu *et al*., 1997, Trounson & Kirby, 1989, Vincent *et al*., 1990). Therefore, advances in assisted reproductive technology have created new possibilities for the prevention and treatment of infertility. Among these methods, ovarian tissue cryopreservation appears to be the most promising—and, perhaps, the only one feasible for prepubertal use, with encouraging results (Binart & Sauvat, 2011). Although the exact number of women who have received autotransplantation of preserved ovarian tissue worldwide is unknown, this procedure has led to 13 reported live births of healthy infants (Donnez *et al*., 2011). These encouraging findings notwithstanding, human ovarian cortex transplantation still carries major limitations. One substantial concern is the possibility of transferring malignant cells back into the patient. Indeed, as ovarian biopsy and cryopreservation is performed before administration of radiation or chemotherapy, there is a risk of ovarian involvement and subsequent reintroduction of the malignancy after autotransplantation. In a retrospective Japanese study performed on autopsy samples, 22.4% of female cancer patients under the age of 40 had ovarian metastases (Kyono *et al*., 2010). More recently, the safety of autotransplantation of ovarian tissue in patients with ovarian tumors was investigated using xenotransplantation into SCID mice. After 24 weeks, there were no gross or microscopic signs of malignancy (Lotz *et al*., 2011). Hence, research interest is now focused on the banking of immature germ cells (Donnez *et al*., 2004, Gosden, 2000, Schroeder & Eppig, 1989). Ovarian tissue cryopreservation provides the possibility of preserving not only reproductive function but also endogenous sex steroid production, thus eliminating the need for later hormone replacement therapy and improving longterm quality of life in this population (Rosa e Silva, 2006).

## MATERIALS AND METHODS

### Animals

The study sample comprised 20 female Wistar rats, approximately 8-12 weeks old. The rat animal model was selected due to the gross similarity between the reproductive systems of human beings and other vertebrates (Mahajan, 1988). In the ovary, the biochemical and physiologic processes are similar in the formation of germ cells, the development of primordial follicles, their subsequent growth to Graafian follicles, eventual ovulation, and anatomic structure.

Although the rat is a polytocous rodent, the female has a rapid regular ovarian cyclicity of 4 to 5 days, making it a good model for studying histologic changes during cryopreservation. Animals were kept on a 12-hour light/dark cycle with a controlled temperature of 22±2°C and were provided food (standard rat chow) and water ad libitum, in accordance with Brazilian National Health Council Resolution 196/96. Bilateral oophorectomy was performed immediately after euthanizing by CO_2_ inhalation.

All procedures were performed in accordance with the Guide for the care and use of laboratory animals (NIH publication no 80-23, revised 1996), in accordance with Biosafety Brazilian Law N° 11.794, 2008 and with prior approval of the local Animal Research Ethics Committee (CEUA/HCPA) under the number GPPG 10-0255.

### Ovary collection

After bilateral oophorectomy, ovaries were bisected, rinsed with PBS to remove excess blood, and placed in human tubal fluid (HTF) embryo culture medium for 2 min. Next, part of the material was placed in 400 µL of 1.5 M DMSO in HTF and the remaining material was placed in in 400 µL of 1,5 M EG in HTF for 15 min at room temperature. Control group ovaries were merely rinsed with PBS to remove excess blood and placed in 10% buffered formalin.

### Cryopreservation

After incubation with DMSO or EG in cryovials, tissue fragments were placed in a Freeze Control® automatic, controlled-rate cryopreservation system, alternately set to one of two cooling curves: curve 1, a slow curve (1 h 50 min), and curve 2, a rapid curve (35 min). Both curves were set to a temperature range of 20°C to -45°C.

Each fresh ovary was subdivided into four fragments. Each sample was submitted to one of the cryoprotectants (DMSO or EG) and cryopreserved under two different freezing conditions, denominated as curve 1 or curve 2. Frozen samples were then placed directly in liquid nitrogen at -196°C.

### Recovery

Specimens were thawed 60 days after freezing. After removal from the liquid nitrogen bath, specimens were kept at room temperature for 2 min, placed in a 37°C ice water bath for 2 min to melt, and then rinsed with HTF to remove the freezing solution. Immediately afterwards, specimens were fixed in 10% buffered formalin at room temperature for over 6 h before paraffin embedding.

### Follicle analysis

Histological analyses were performed with hematoxylin & eosin-stained, 4-µm-sliced, paraffin-embedded samples. Follicles were assessed by light microscopy (400x magnification) and classified according to the modified Oktay *et al*. (1995) criteria.

In this classification scheme, preantral follicles are classified as follows according to developmental stage: primordial, when exhibiting one layer of sparse, rectangular (flat) pre-granulosa cells surrounding the oocyte; or primary, when exhibiting at least one layer of cuboid or columnar pre-granulosa cells, until the oocyte is encapsulated by a single layer of cuboid granulosa cells.

Follicles were examined and classified as preserved or not preserved.

Follicles with cytoplasmic vacuolization or an irregular cell outline were still classified as preserved, as these changes are considered reversible.

Conversely, follicles exhibiting pyknosis were considered damaged. To evaluate ovarian functionality, primordial and primary follicles were counted in the total area of each ovarian slice.

### Statistical analysis

The effects of cryopreservation on follicular integrity were assessed by ANOVA. Tukey’s test was used for comparison between means. The significance level was set at 5% (*P*<0.05) for all analyses.

## RESULTS

**Figure 1:**
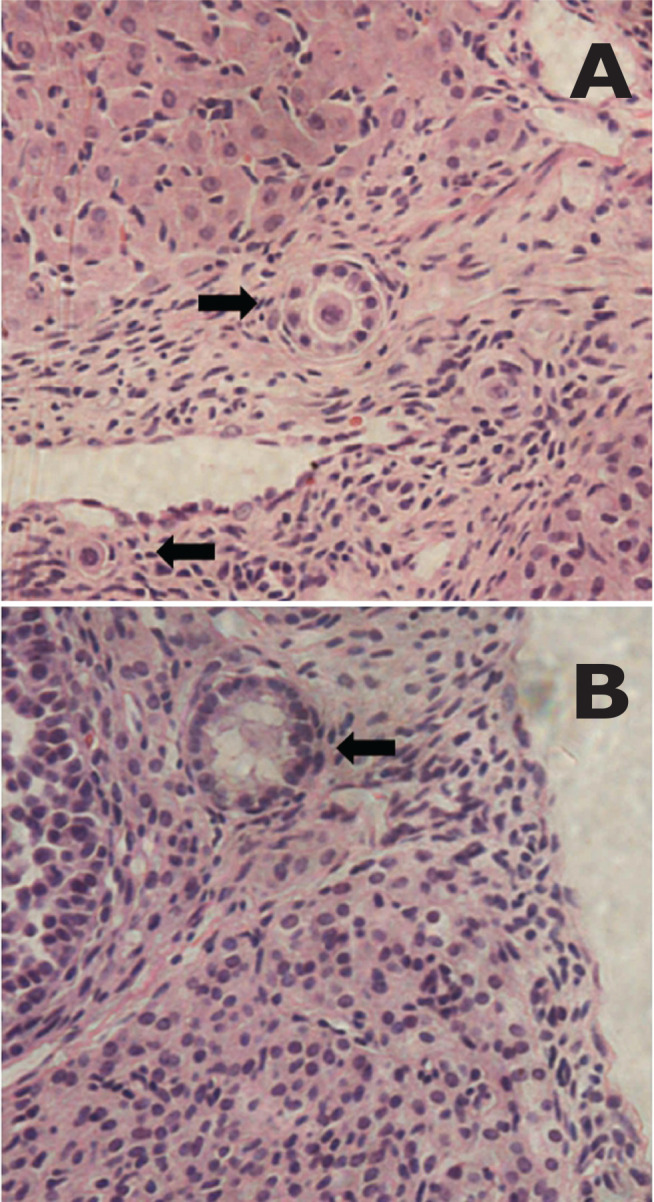
Photomicrograph of cryopreserved ovarian tissue **A.** Superior arrow shows a normal primary follicle and inferior shows a normal primordial follicle. **B.** Arrow shows pyknotic primary follicle.

In hematoxylin & eosin-stained sections corresponding to the control group (non-frozen tissue), all follicle types were present (primordial, primary, secondary, preantral, and antral), whereas only primordial and primary follicles were present in cryopreserved sections (Figure 1A).

The most frequent morphological changes in cryopreserved tissue were cytoplasmic vacuolization and irregular cell outline (Figure 1B).

In cryopreserved tissue, the follicles with preserved integrity in each ovary were 79% primordial and 29% primary. Microscopy analysis of cryopreserved ovarian tissue after thawing showed that use of EG as a freezing solution leads to a greater number of viable primordial and primary folicles on recovery compared to DMSO (P<0.05).

**Figure 2:**
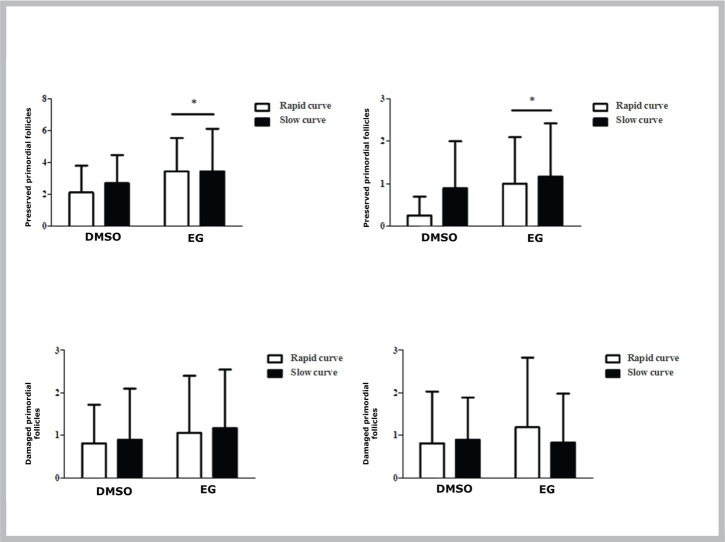
Follicular integrity Follicular integrity (mean ± standard deviation) after cryopreservation of ovarian tissue with two different freezing solutions (DMSO or EG) and using two different cooling curves (rapid or slow).

Comparison of tissues exposed to the two different cooling curves (slow versus rapid freezing) revealed no statistically significant differences in the number of viable primordial and primary follicles (*P*>0.05) (Figure 2).

## DISCUSSION

Several studies have focused on improving techniques for cryopreservation of reproductive tissue, with particular emphasis on preserving the integrity of ovarian tissue. The purpose of the present study was to assess the effects of freezing solutions with two different cryoprotectants (EG and DMSO) combined with two different cooling curves, one rapid and one slow, on an automated cryopreservation system.

Efficiency in ovarian tissue cryopreservation was defined as the number of preserved primordial and primary ovarian follicles, which are potentially capable of embryo generation after in vitro fertilization if adequate conditions are met. In this study, a greater number of viable primordial and primary follicles were recoverable from tissue preserved with the EG-based freezing solution, regardless of which cooling curve was used during cryopreservation. This result was most likely due to the lower embryonic cell toxicity and higher cell membrane permeability of EG (Miyamoto & Ishibashi, 1978).

Lucci *et al*. (2004) compared cryopreservation of four different cryoprotectants (PROH, GLY, DMSO, EG) at different strengths in Bos indicus ovarian tissue. More effective outcomes were obtained with DMSO- and PROH-based freezing solutions, which preserved the structural integrity of somatic and germ cells alike. This divergence from the results of the present study may have been due to interspecies differences in ovarian tissue; perhaps bovine follicles are more sensitive to the toxic effects of EG than those of other species. In prior studies, EG was shown to be nontoxic to human (Chi *et al*., 2002) and bovine embryos (Sommerfeld & Niemann, 1999, Visintin *et al*., 2002). Furthermore, use of EG was shown to be more effective in the cryopreservation of murine ovarian tissue, with 88% of follicles remaining morphologically normal after thaw (Candy *et al*., 1997); however, the authors warned that prolonged exposure to EG might decrease follicular viability. In another study of human ovarian tissue, 84% of follicles survived after cryopreservation in EG (Newton *et al*., 1996). These findings are consistent with the results of the present experiment: EG was found to be less toxic as a freezing solution, as shown by its superior preservation of the structural integrity of ovarian tissues. However, Candy *et al*. (1997) noted that prolonged exposure of rat ovaries to EG prior to freezing may lead to follicular toxicity due to the high cell membrane permeability of EG. Lucci *et al*. (2004) reported that, overall, the phase of follicle development (primordial, primary, or secondary) did not influence follicular resistance to cryoprotectants.

There were no statistically significant differences in the efficacy of the cooling curves used in this study. This result indicates that, for conventional freezing, the conventional freezing process can be shortened without damaging cryopreserved tissue.

Several authors have proposed slow cooling techniques, as ovarian tissue is highly tolerant to the cryopreservation process (Gosden, 2000, Hovatta *et al*., 1996, Newton *et al*., 1996). Most studies have compared conventional freezing with vitrification. Isachenko *et al*. (2009) tested vitrification versus conventional freezing of human ovarian tissue and concluded that conventional freezing is the more promising technique, as tissue thus preserved conserves a higher development potential.

The authors believe this may be due to contamination of liquid nitrogen, which is used in freezing and storage and is susceptible to microbial contamination (Bielanski *et al*., 2000, Tedder *et al*., 1995). In a recent study, good results were obtained with the vitrification protocol previously described by Kagawa *et al*. (2009) and Silber (2012) in combination with the metallic grid vitrification technique (Herraiz *et al*. 2014). Using immunohistochemical analysis with Ki-67, Ceschin *et al*. (2006) concluded that although both conventional freezing and vitrification were feasible methods for ovarian tissue cryopreservation, vitrification was associated with the recovery of a greater number of potentially viable primordial follicles in rats.

Overall, a good cell freezing protocol is one that ensures the stability of cross-reactions between molecules in the intracellular environment and enables cell survival after thawing. Viable tissues should not only permit cell survival but also exhibit similar baseline architectures, including cell junctions, interactions, and molecular bonds (Jones & Darville, 1989). Cryobiology studies have shown that different types of cells exhibit different thawing behaviors even when stored in the same freezing solution. Due to the high complexity of ovarian cell architecture, survival of cryopreserved tissue depends not only on freezing and thawing curves but also on the method used for removal of cryoprotectants (Jones & Darville, 1989).

## CONCLUSION

In this study, regardless of the cooling curve, EG was superior to DMSO as a cryoprotectant for cryopreservation of ovarian tissue in the Wistar rat, as it was associated with a greater number of potentially viable primordial and primary follicles on histological analysis. As there was no significant difference between cooling curves, after histological evaluation one may conclude that conventional rapid freezing protocols produced a superior number of viable primordial and primary follicles obtained from rat cryopreserved ovary tissues.
